# Effect of Elevation Training Mask on Swallowing Function in Individuals with Parkinson’s Disease

**DOI:** 10.1007/s00455-025-10815-5

**Published:** 2025-02-25

**Authors:** Yuval Nachalon, Dina Shpunt, Anat Zait, Yael Oestreicher-Kedem, Liav Hayat, Yarden Ashkenazi, Nogah Nativ-Zeltzer, Peter C. Belafsky, Gadi Maayan Eshed, Tanya Gurevich, Yael Manor

**Affiliations:** 1 Department of Otolaryngology, Head and Neck Surgery and Maxillofacial Surgery, Tel-Aviv Sourasky Medical Center, 6 Weizman Street, Tel-Aviv, Israel; 2 Movement Disorders Unit, Dept of Neurology, Tel Aviv Sourasky Medical Center, Tel-Aviv, Israel; 3 Faculty of Medical and Health Sciences, Tel Aviv University, Tel Aviv, Israel; 4 Faculty of Health Professions, Communication Sciences and Disorders Department, Ono Academic College, Kiryat Ono, Israel; 5 Department of Otolaryngology-Head and Neck Surgery, University of California Davis, Sacramento, CA USA

**Keywords:** Parkinson’s disease, Elevation training mask (ETM), Swallowing dysfunction, Respiratory muscle strengthening, Fiberoptic endoscopic evaluation of swallowing (FEES)

## Abstract

To evaluate the impact of the elevation training mask (ETM) on swallowing safety and swallowing efficiency in patients with Parkinson’s disease (PWP) when used as a respiratory muscle strengthening tool. Study Design. Prospective cohort study. Setting. Tertiary university-affiliated medical center. Thirteen PWP underwent Fiberoptic Endoscopic Evaluation of Swallowing and spirometry assessments both before and after a 4-week ETM use, which included incrementally increasing resistance each week. Measurements taken included EAT-10, swallowing disturbances questionnaire (SDQ), Penetration Aspiration Score (PAS), Yale Pharyngeal Residue Severity Rating Scale, and Peak Expiratory Flow (PEF). Disease severity was assessed using the Unified Parkinson’s Disease Rating Scale (UPDRS). Eleven out of 13 male participants (median age 70 years, UPDRS 33, disease duration 8.5 years) completed the 4-week protocol (84.6% completion rate). Vallecular residue significantly decreased for solids (median from 3.0 [IQR: 2.0–3.0] to 2.0 [IQR: 1.0–2.0], p = 0.028) and semi-solids (from 3.0 [IQR: 2.0–4.0] to 2.0 [IQR: 1.0–3.0], p = 0.025), with a non-significant improvement for liquids (from 2.0 [IQR: 2.0–2.0] to 2.0 [IQR: 1.0–2.0], p = 0.19). Patient-reported outcomes (EAT-10, SDQ, VHI-10, RSI) and PEF showed non-significant trends toward improvement. A 4-week use of ETM, serving as a form of respiratory muscle strengthening, demonstrated specific improvements in vallecular residue for semi-solid and solid consistencies in PWP with dysphagia. While other swallowing and respiratory measures showed positive trends, these changes did not reach statistical significance. Further research with a larger cohort is needed to evaluate ETM’s role in swallowing rehabilitation.

## Introduction

Dysphagia poses significant challenges for patients with Parkinson’s disease (PWP), leading to various complications including dehydration, malnutrition, and increased risk of aspiration pneumonia, ultimately contributing to decreased quality of life and mortality. [1] Swallowing disorders are particularly prevalent in this population, representing one of the leading causes of death among PWP. [2, 3] Recent guidelines and systematic reviews have highlighted the complexity and multifaceted nature of dysphagia management in PWP, emphasizing the need for comprehensive treatment approaches.[4–6] Variability in outcomes and measures across dysphagia trials in Parkinson’s disease (PD) makes it difficult to compare interventions. Patient, caregiver, and clinician perspectives are often overlooked in defining meaningful outcomes. The Core Outcome Set for Dysphagia Interventions in Parkinson’s Disease (COS-DIP) seeks to standardize patient-centered outcomes and improve research relevance and comparability [7].

The etiology of dysphagia in PD is multifactorial, involving disruptions across all phases of swallowing, including reduced mastication speed and coordination, pharyngeal muscle atrophy, esophageal dysmotility, sensory deficits, and impaired cough reflex. [8–10] Effective management of dysphagia in PD requires a comprehensive approach addressing both sensory and motor components of swallowing, often supplemented with pharmacological interventions. Given the fluctuating nature of dysphagia in PWP, optimization of medication dosage is crucial as well for enhancing swallowing safety, particularly during off-states. [11].

Dysphagia treatment by a speech language pathologist (SLP) can vary and should be tailored to the patient’s medical and cognitive state and dysphagia severity. Traditional swallowing therapy have encompassed a range of strategies such as environmental modifications, oral motor exercises, dietary modifications, swallowing postures and maneuvers aimed to improve and maintain swallowing function and safety in PWP [12]. Additionally, emerging evidence suggests a potential role for respiratory muscle strength training (RMST) in enhancing swallowing function and airway protection [13, 14]. It involves strengthening of the inspiratory or expiratory muscles, both have been investigated in PWP [15]. Inspiratory muscle strength training (IMST) was found to improve dyspnea and inspiratory muscle strength [16].

EMST was initially designed to target PWP who experience swallowing difficulties, aiming to enhance the force generation capacity of pharyngeal and suprahyoid muscles [15, 17, 18]. Studies have consistently shown that EMST, either used alone or in conjunction with other therapeutic approaches, leads to enhancements in expiratory flow, reductions in Penetration-Aspiration Scale (PAS) scores, and increases in hyoid displacement during swallowing.[13, 19]. A randomized controlled trial comparing EMST with sensorimotor training for airway protection (smTAP) demonstrated that smTAP provides greater improvements in reflex cough peak expiratory flow, expired volume, and urge to cough compared to EMST, which primarily enhances maximum expiratory pressure. [20] This suggests that interventions targeting both strength and skill-based mechanisms may be more effective in addressing the multifaceted nature of airway protection deficits in PWP [21].

A variety of investigations have explored the potential mechanisms underlying the beneficial effects of EMST on swallowing rehabilitation. Electromyography (EMG) studies comparing suprahyoid muscle activity during swallowing pre/post EMST have demonstrated prolonged activity and increased amplitude, indicative of increase in motor unit activation. Additionally, ultrasound assessments have revealed an augmentation in geniohyoid muscle mass pre/post EMST, contributing to enhanced hyoid elevation.[22, 23] High-resolution manometry and EMG analyses conducted during EMST have noted elevated velopharyngeal closing pressure and increased palatal/pharyngeal EMG activity correlating with escalating expiratory load.[24] The stimulation of suprahyoid muscles during the pharyngeal phase of swallowing promotes superior-anterior movement of the hyo-laryngeal complex, thereby facilitating upper esophageal sphincter (UES) opening and improving airway protection. These findings suggest that EMST may indirectly enhance pharyngeal muscle strength through its effects on hyolaryngeal elevation and associated mechanisms, such as bolus transition and airway protection. This may explain the observed improvements in swallowing function following EMST, though the exact mechanisms require further study.EMST was found more beneficial in PWP with swallowing disturbances compared to IMST.[14, 17, 25].

Elevation training mask (ETM) is a device designed to simulate altitude training. It is worn over the mouth and nose and has adjustable valve featuring six resistance levels. The valve restricts the flow of air on both inhalation and exhalation, necessitating increased effort from respiratory muscles to ensure adequate respiration similar to RMST. In a previous pre-clinical study Shih and Belafsky demonstrated that ETM provides adjustable expiratory muscle strength resistance pressures and suggested that it can serve as an effective treatment modality for pulmonary dysfunction and swallowing disorders through RMST.[20].

The purpose of the current study was to evaluate the impact of the ETM on swallowing of PWP when used as a respiratory muscle strengthening tool.

## Methodology

The study was approved by the institutional review board. Thirteen males were recruited to the study, all were diagnosed with neurodegenerative disease, idiopathic PD. Their genetic condition is unknown for the researchers. All participants underwent Fiberoptic Endoscopic Evaluation of Swallowing (FEES) and spirometry assessments both before and after a 4-week ETM training program. Right after the initial FEES, participants completed several questionnaires, including the Eating Assessment Tool-10 (EAT-10), Reflux Symptom Index (RSI), Voice Handicap Index (VHI-10) and Swallowing Disturbance Questionnaire (SDQ) and underwent the Unified Parkinson’s Rating Scale (UPDRS) by a neurologist. [26–30] The RSI was included as it evaluates laryngeal symptoms commonly associated with swallowing disorders. After the initial FEES assessment, if the participants were deemed eligible for study participation, a speech-language pathologist (SLP) not involved in the swallowing evaluation provided instruction on the proper use of the ETM. Inclusion criteria were PWP with oropharyngeal swallowing disorder as confirmed by FEES (specifically including those with pharyngeal residue), capability to comply with study requirements, and willingness to participate in both virtual and face-to-face meetings with the SLP who provided swallowing therapy. Exclusion criteria included individuals with severe cognitive impairment, significant comorbidities, prior head or neck surgery, or those requiring oxygen therapy or inhaler use. Additionally, participants currently undergoing treatment for swallowing disorders or communication barriers were excluded from the study.

### Fiberoptic Endoscopic Evaluation of Swallowing (FEES) Assessment

The FEES assessment utilized a video laryngoscope (VNL11-J10, PENTAX Europe GmbH, Hamburg, Germany). The laryngoscope was inserted through the nose without the application of a topical anesthetic or vasoconstrictor to the nasal mucosa and positioned in either the nasopharynx or oropharynx. Participants then underwent various swallowing tasks, including consuming colored water (International Dysphagia Diet Standardization Initiative (IDDSI) 0) in specific volumes (two 5 ml, two 10 ml, two free sips from a cup, and continuous sips of 3–4 swallows through a straw), followed by applesauce (IDDSI 4; two spoons of 5 ml) and bites of a biscuit (IDDSI7; two bites).[31] After each swallow, the pharynx was examined for residue in the vallecula and the pyriform sinus, with grading performed according to the Yale Pharyngeal Residue Severity Rating Scale.[32] If residue was present, participants were prompted to swallow again to clear it, with additional swallows provided if strategies were employed. Aspiration and penetration were assessed after each swallow and graded using the PAS score.[33] The assessments were conducted by a trained laryngologist and a SLP, with the latter blinded to the participants’ mask usage status. For statistical analysis we udes: for IDDSI Level 0, the mean of four sips was calculated (10 ml and free sips). For IDDSI Levels 4 and 7, the mean of two swallows was calculated for each participant.

### Spirometry Assessment

Peak expiratory flow (PEF) measurements were conducted using an electronic spirometer (MiniSpir, MIR Medical International; Research S.r.l., Rome, Italy). Participants were instructed to breathe in as much as they could, insert the mouthpiece, seal their lips tightly around it and exhale forcefully. The procedure was repeated three times, and the highest score was recorded. PEF measurements were taken both before and 4 weeks after the use of the ETM.

### RMST Protocol using ETM

**Fig. 1:**
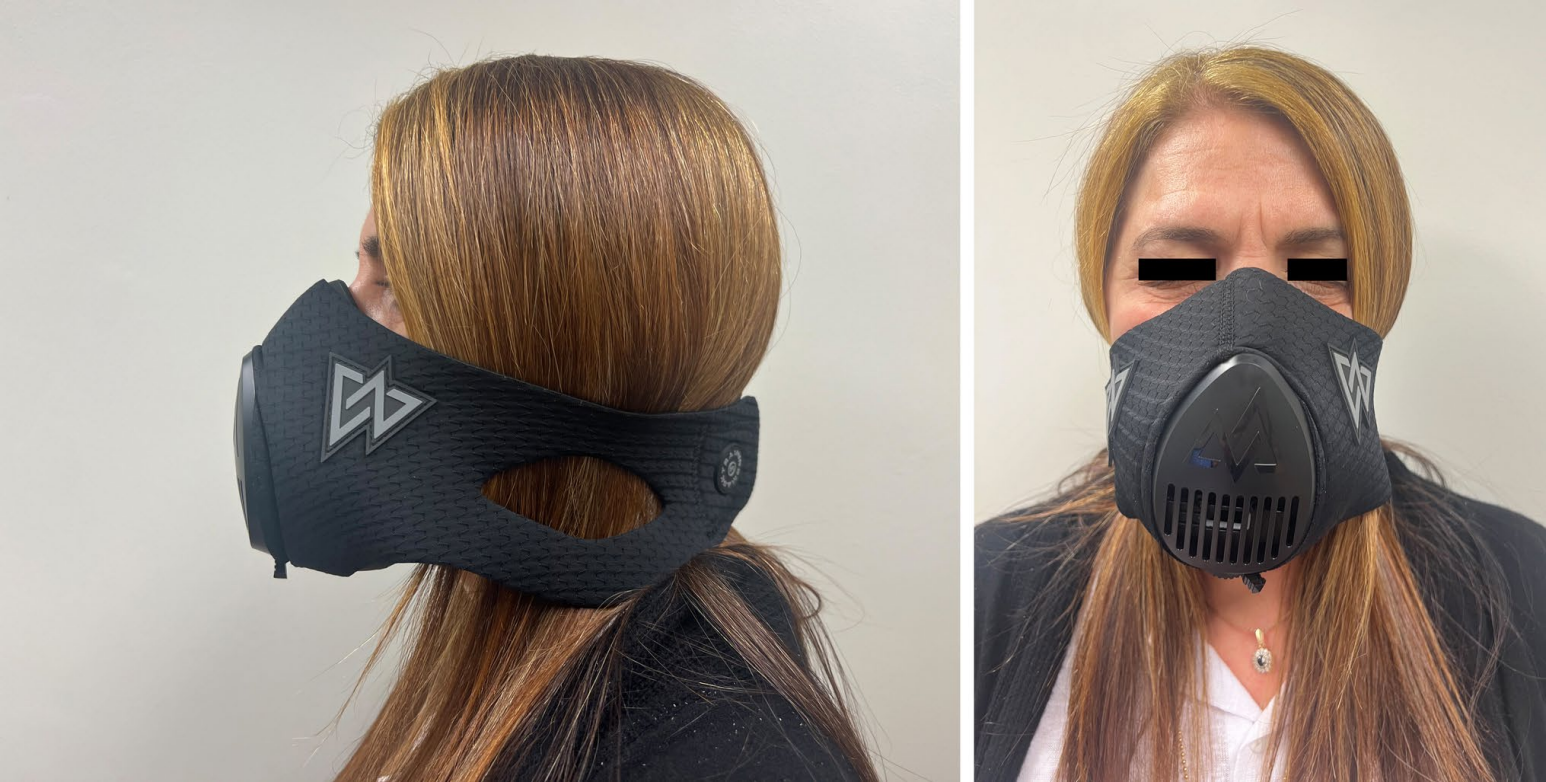
Lateral and front view of the Elevation Training Mask 3.0

Participants were provided with ETM 3.0 (Training Mask LLC, Cadillac, MI) and were directed to wear it four times daily for five-minute intervals for five days per week. (Fig. 1) They were instructed to progressively enhance resistance over the course of the intervention period by gradually closing the valve until it reached full closure by the fourth week. The mask featured six resistance levels, prompting patients to elevate resistance by two levels during the initial two weeks, followed by increments of one level per subsequent week. Once a week, participants engaged in an online session with the SLP, during which they increased resistance level and wore the mask for 5 min, facilitating observation of their performance by the SLP.

The ETM 3.0 is a resistive apparatus designed to enclose the nose and mouth, forming a sealed system. It is available in three sizes based on user weight and features six adjustable resistance levels controlled by a small knob.

**Fig. 2:**
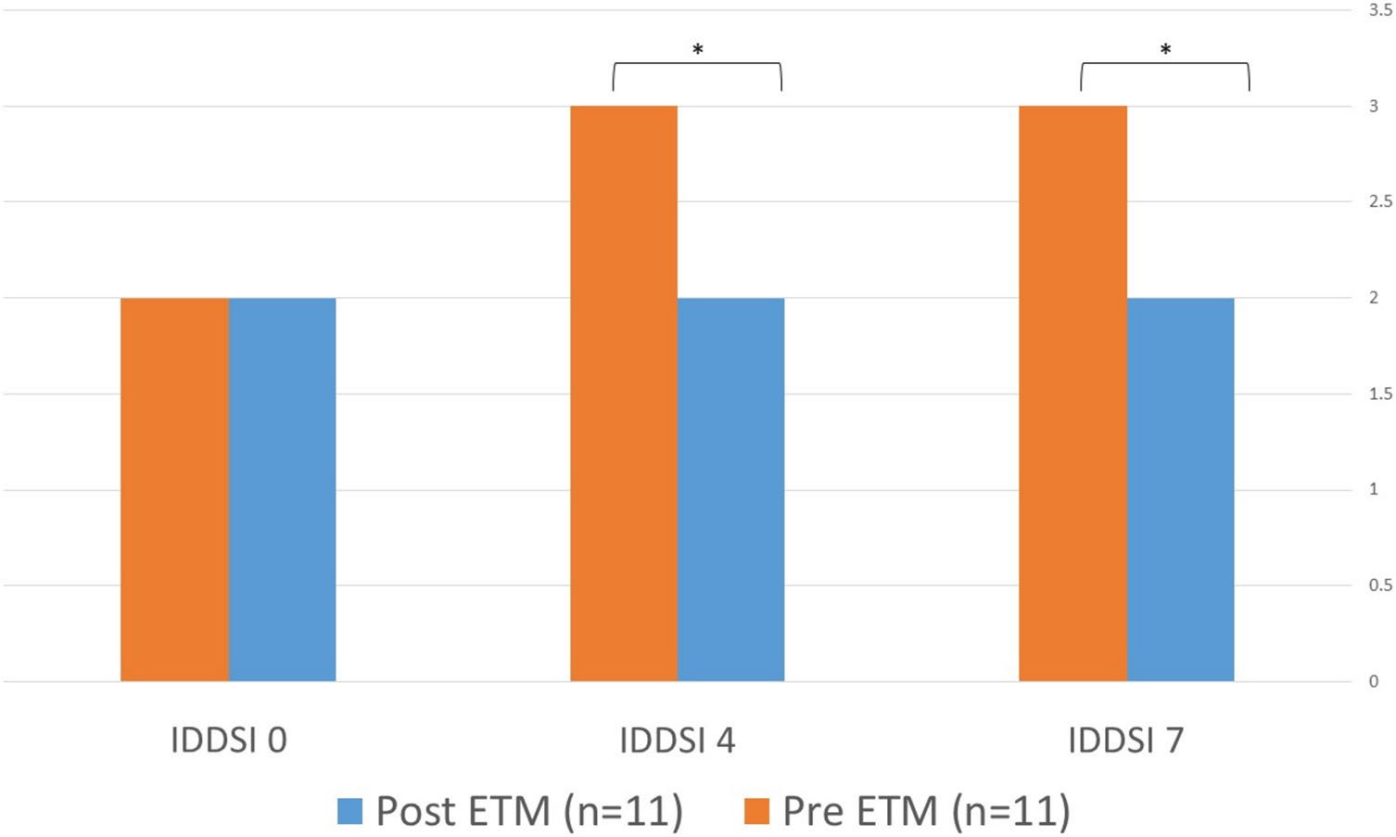
Yale vallecular score pre and post ETM treatment, (p < 0.05)

### Participant Feedback

During weekly online sessions and at the final visit, participants were asked about their experience with the ETM protocol through informal conversations. Notes were taken regarding any difficulties encountered, protocol adherence, and general feedback about the training process.

**Table 1:** Clinical data of individuals before and after ETM treatment, values presented as median (interquartile range)

Measure	Pre ETM (n = 11)	Post ETM (n = 11)	P value
Vallecular Residue—Solid (IDDSI 7)	3.0 (2.0–3.0)	2 (1.0–2.0)	0.028*
Vallecular Residue—Semi-solid (IDDSI 4)	3.0 (2.0–4.0)	2.0 (1.0–3.0)	0.025*
Vallecular Residue – Liquid (IDDSI 0)	2.0 (2.0–2.0)	2.0 (1.0–2.0)	0.19
Peak Expiratory Flow (L/s)	4.5 (3.2–5.8)	4.7 (3.5–5.9)	0.6
Eating Assessment Tool 10 Score	10.0 (6.0–15.0)	8.0 (5.0–13.0)	0.7
Swallowing Disturbances Questionnaire Score	14.0 (9.5–19.0)	12.0 (8.0–17.0)	0.4
Voice Handicap Index 10 Score	20.0 (15.0–28.0)	19.0 (14.0–27.0)	0.2
Reflux Symptom Index Score	17.0 (12.0–23.0)	14.0 (10.0–20.0)	0.4

*IDDSI* International dysphagia diet standardization initiative

### Statistical Analysis

All data were coded and recorded into SPSS Version 25.0 (IBM Inc., Armonk, NY Given our small sample size (n = 13), we employed non-parametric statistics to avoid making assumptions about data distribution. The Wilcoxon signed-rank test was used for pre-post comparisons, as it is more appropriate for small samples and does not require normality assumptions. Results are presented as median and interquartile range (IQR). Statistical significance was set at p < 0.05.

## Results

Among the thirteen male participants initially recruited, eleven completed the full protocol. The median age of completers was 70 years (IQR: 65–75), with median disease severity of 33 (IQR: 25–41) on the UPDRS and median disease duration of 8.5 years (IQR: 4.7–12.3).

FEES examination revealed significant improvements in vallecular residue for both solid and semi-solid consistencies. For solid consistency (IDDSI 7), median residue decreased from 3.0 (IQR: 2.0–3.0) to 2.0 (IQR: 1.0–2.0, p = 0.028). Semi-solid consistency (IDDSI 4) showed improvement from 3.0 (IQR: 2.0–4.0) to 2.0 (IQR: 1.0–3.0, p = 0.025). Liquid consistency (IDDSI 0) showed a non-significant trend toward improvement from 2.0 (IQR: 2.0–2.0) to 2.0 (IQR: 1.0–2.0, p = 0.19) (Fig. 2).

Penetration-Aspiration Scale scores showed non-significant improvements across all consistencies. For solid consistency, median scores decreased from 1.0 (IQR: 1.0–1.0) to 0.9 (IQR: 0.0–1.0, p = 0.30). Semi-solid consistency showed a decrease from 1.15 (IQR: 1.0–1.5) to 0.88 (IQR: 0.5–1.0,p = 0.30), while liquid consistency decreased from 2.75 (IQR: 1.0–4.0) to 1.1 (IQR: 1.0–2.0, p = 0.10).

Peak expiratory flow measurements showed minimal change, with median values increasing from 4.5 L/s (IQR: 3.2–5.8) to 4.7 L/s (IQR: 3.5–5.9, p = 0.60).

Analysis of patient-reported outcomes revealed non-significant trends toward improvement across all measures. EAT-10 scores showed a median decrease from 10.0 (IQR: 6.0–15.0) to 8.0 (IQR: 5.0–13.0, p = 0.70). SDQ scores decreased from 14.0 (IQR: 9.5–19.0) to 12.0 (IQR: 8.0–17.0, p = 0.40). VHI-10 scores changed from 20.0 (IQR: 15.0–28.0) to 19.0 (IQR: 14.0–27.0, p = 0.20), and RSI scores decreased from 17.0 (IQR: 12.0–23.0) to 14.0 (IQR: 10.0–20.0, p = 0.40) (Table 1).

During follow-up conversations, participants shared their experiences with the protocol. Several reported that the final week was particularly challenging due to the maximum resistance level. Protocol adherence was a challenge, with some participants reporting difficulty remembering to complete four daily sessions. As a result, they relied on spouse or caregiver assistance to maintain the exercise schedule. Two participants withdrew from the study: one declined to perform the post-therapy FEES, and the other was unable to complete the therapy protocol due to health issues.

## Discussion

This study assessed the potential use of ETM as a respiratory muscle strengthening tool on swallowing function in PWP. Our findings suggest an improvement in vallecular residue for semi-solid and solid consistencies, with other swallowing and respiratory parameters showing non-significant trends toward improvement. Overall, the treatment appears to be well tolerated with an 84.6% completion rate (11/13 participants), although some participants needed assistance from caregivers. Dysphagia in PWP encompasses disruptions across all phases of swallowing. Prevalent findings in PWP include oropharyngeal bradykinesia, incoordination, and reduced anterior hyoid bone movement.[34, 35] Fattori et al. conducted pharyngeal high-resolution manometry and reported a 66% reduction in tongue base pressure in PWP compared to healthy adults. [36] This reduction may explain the higher vallecular residue detected by FEES in our cohort before rehabilitation. However, vallecular residue can result from a combination of factors other than tongue weakness, such as impaired pharyngeal contraction, reduced laryngeal elevation, and muscle coordination impairment.

EMST was developed specifically to treat PWP with swallowing disturbances.[17, 18] While the goal of EMST is to improve hyoid and larynx elevation which may subsequently lead to enhanced pulmonary function, cough effectiveness and swallowing functions, the exact mechanism of improvement requires further investigation. Mechanistic investigations have suggested that EMST enhances pharyngeal muscle strength, bolus transition, and airway protection through heightened motor unit activation and increased geniohyoid muscle mass.[22–24] Our study demonstrated that 4 weeks of ETM use led to significant improvements in vallecular residue, particularly for solid consistencies where median scores improved from 3.0 to 2.0 (p = 0.028), and semi-solid consistencies where median scores improved from 3.0 to 2.0 (p = 0.025). This differential response may be explained by the higher level of muscular demands in managing solid boluses. The lack of significant improvement in liquid residue and PAS might be due to lower baseline scores, suggesting a floor effect. Another option is that, while vallecular residue and penetration-aspiration are both aspects of swallowing dysfunction, they are related to different physiological mechanisms. The ETM protocol may more directly impact the muscle strength needed for bolus clearance than the temporal coordination required for airway protection. The lack of significant changes in patient-reported outcomes, despite observable improvements on FEES, raises important considerations. This discrepancy may reflect a delay between objective improvements and patients’ subjective awareness of changes, the need for a longer intervention period to achieve perceptible symptomatic relief, or the possibility that reductions in vallecular residue alone do not meaningfully impact overall swallowing-related quality of life.EMST is widely utilized in the rehabilitation of PWP.[15] Several instruments with similar principles are available today, typically involving a small tube with adjustable resistance levels. Selecting the right instrument for the patient is crucial, as it requires adequate lip strength and coordination. Additionally, medical provider assistance is often necessary to set up the instrument. Our experience with ETM suggests several practical advantages: ETMs are worn over the face, targeting both the mouth and nose, without the need for manual support or lip seal. Setting the mask is straightforward, allowing patients to be independent in the process and receive remote instructions. Furthermore, ETMs are cost-effective and can be worn during daily activities and during monitored occupational and physical therapy rehabilitation tasks, even in individuals with cognitive decline. However, our findings indicate that caregiver support may still be beneficial for maintaining exercise adherence, particularly during the final week of progressive resistance training.

This study has several limitations. The small sample size (n = 11) limits statistical power, and the homogeneity of the population, with all participants being male, limits the generalizability of the findings. The absence of a control group and the single post-treatment assessment further limit the study’s design. We tried to mitigate the impact of the small cohort size by recruiting only male participants with moderate dysphagia symptoms in order to maintain a more homogenous sample and reduce variability, and the participants did not undergo any other rehabilitative treatment during the study period. Including patients with severe dysphagia would have raised ethical concerns, as withholding necessary interventions during the study would not have been appropriate.

Larger, controlled trials with more diverse populations will help to increase the generalizability of the findings. Longer follow-up periods and changes to the training protocols would help assess the durability of effects over time and the optimal resistance progression and training protocols to maximize impropvement.

## Conclusion

A 4-week use of ETM demonstrated significant improvements in vallecular residue for solid and semi-solid consistencies in PWP with dysphagia, with good protocol tolerability (84.6% completion rate). While these findings are promising, larger controlled studies are needed to comprehensively evaluate ETM’s role in swallowing rehabilitation and determine optimal patient selection criteria.
